# Simulation of the Urban Space Thermal Environment Based on Computational Fluid Dynamics: A Comprehensive Review

**DOI:** 10.3390/s21206898

**Published:** 2021-10-18

**Authors:** Hongyuan Huo, Fei Chen, Xiaowei Geng, Jing Tao, Zhansheng Liu, Wenzhi Zhang, Pei Leng

**Affiliations:** 1 Faculty of Architecture, Transportation and Civil Engineering, Beijing University of Technology, Beijing 100124, China; huohongyuan@bjut.edu.cn (H.H.); s201904037@emails.bjut.edu.cn (F.C.); gengxw@emails.bjut.edu.cn (X.G.); taoj@emails.bjut.edu.cn (J.T.); liuzhansheng@bjut.edu.cn (Z.L.); 2 School of Geosciences and Resources, China University of Geosciences (Beijing), Beijing 100083, China; 2001210201@email.cugb.edu.cn; 3 Institute of Agricultural Resources and Regional Planning, Chinese Academy of Agricultural Sciences, Beijing 100081, China

**Keywords:** computational fluid dynamics, urban space thermal environment, urban heat island, urban landscape, remote sensing

## Abstract

Rapid urbanization has made urban space thermal environment (USTE) problems increasingly prominent. USTE research is important for improving urban ecological environment and building energy consumption. Most studies on USTE research progress have focused on meteorological observations and remote sensing methods, and few studies on USTE are based on computational fluid dynamics (CFD). During the past two decades, with the increasing applications of CFD in USTE research, comprehensively summarizing the phased results have become necessary tasks. This paper analyzes the current research status of CFD-based USTE simulation from six perspectives. First, we summarize the current research status of USTE simulation with CFD models that integrate ground observations and remote sensing technology. Second, we define and classify the spatial scope of CFD-based USTE simulations at different scales. Third, we systematically analyze the quantitative relationships among urban land type, the underlying surface structure, water bodies, green space and the corresponding changes in CFD-based USTE simulations. Fourth, we quantitatively analyze the impact of anthropogenic heat in CFD-based USTE simulations. Fifth, we summarize the corresponding USTE mitigation measures and methods based on the CFD simulation results. Finally, the outlooks and the existing problems in current research on CFD simulations of the USTE are analyzed.

## 1. Introduction

In recent years, with the acceleration of urbanization and the rapid development of industry, populations have been migrating to cities [1], resulting in various urban ecological and environmental problems [2]. Among them, the urban thermal environment has become particularly prominent, attracting widespread attention from many scientists [3,4]. Urban thermal environment issues affect people’s living comfort, the urban climate, the atmospheric environment, and biological habits, often lead to enhanced energy consumption and greenhouse gas emissions, and increase the incidence and mortality of thermal environment-related diseases [5].

The urban space thermal environment (USTE) is spatially expressed as the horizontal and vertical distributions of the surface temperature and atmospheric temperature fields [6]. With the urban space temperature field as the core, the USTE is the physical environmental system in which the underlying surface, atmospheric transmission and solar radiation are influenced by humans and their interactions with nature [7,8,9,10]. The urban thermal environment has significant impacts on the urban climate and micrometeorology; it is an important indicator used to measure the state of the urban ecological environment, and its temporal and spatial evolution processes are closely related to societal and economic activities [11,12]. Therefore, studying the USTE and spatially quantitatively analyzing the distribution of the urban spatial temperature field, considering the effects of temporally and spatially varying processes, is of great importance to urban ecological security and sustainable development.

The current methods for quantitatively studying three-dimensional USTEs include ground observation, remote sensing, and numerical simulation [13]. USTE research based on ground observations has provided effective estimates of the distribution and spatiotemporal dynamics of station temperatures at different time scales, but the spatial representation of these discretely distributed weather stations is poor. Although USTE research based on remote sensing can retrieve the surface temperature field and the surface ecological parameters, the heat transfer among different underlying surfaces is not considered. Numerical simulation can be used for the three-dimensional simulation of USTEs [5,14]. Computational fluid dynamics (CFD) is one of the most commonly used numerical simulation methods and can be used to simulate the motion of turbulent fluids. Combining the advantages of fluid mechanics and heat transfer, CFD can simulate the physical processes of heat conduction and heat convection among underlying surfaces in a city at a fine scale [15].

**Figure 1:**
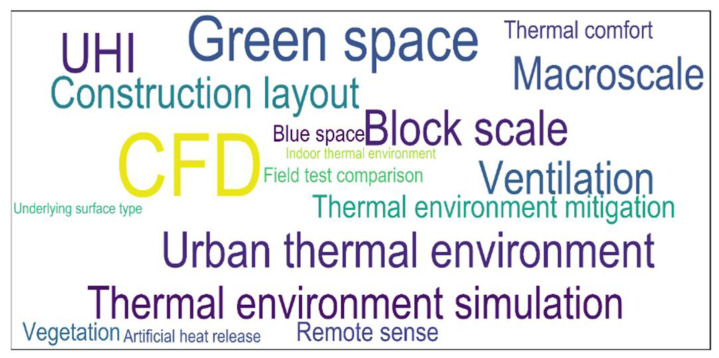
Word cloud diagram of keywords from CFD-based thermal environment research.

Recently, CFD technology has been effectively applied for quantitative research and simulations of USTEs. The literature from the past two decades demonstrates the considerable progress that has been made by many scientists, who have obtained valuable results [15]. From the perspective of the currently published literature, the word cloud diagram shown in Figure 1 was obtained by counting the keywords in studies that used CFD technology to study USTEs. From Figure 1, most researchers of the thermal environment focused on mitigation measures and simulated the thermal environment with CFD technology; additionally, the relationship between the urban landscape and the USTE was often considered, and USTEs were often evaluated at the block scale or mesoscale by combining CFD and remote sensing technology.

However, few studies have summarized the current status of research on the urban thermal environment based on CFD methods or the integration of CFD and remote sensing. Therefore, a timely review of research on CFD simulations of the USTE is valuable for further understanding the mechanisms of the USTE, improving urban planning and design, mitigating negative changes to the urban thermal environment, and quantitatively evaluating the urban thermal environment and human comfort levels. Thus, we summarize the current research status of CFD-based urban thermal environment problems over the past two decades, the related methods of USTE monitoring based on CFD models, the influential factors and corresponding relationship between CFD and the USTE, and the progress achieved with USTE mitigation measures. Future research directions and research focuses based on CFD methods are proposed.

## 2. CFD-Based Simulation Method for the USTE

CFD simulations of the thermal environment are based on computational numerical simulation theory and methods. Numerical simulation methods mainly include the finite difference method, finite element method, boundary element method, and finite volume method. Simulations of the USTE mainly use the finite element analysis method to solve fluid mechanics models, thereby simulating the spatial thermal environment.

Based on whether this method is combined with other technologies and whether the simulation results are validated or not, this field of research is assessed from four perspectives in this section. First, we introduce the definition of CFD numerical simulation and the corresponding software. Second, we classify and introduce the CFD numerical simulation methods applied to the spatial thermal environment. Third, studies that have verified CFD simulation results based on ground observation data are summarized. Finally, research on CFD coupled with remote sensing, geographic information, laser scanning and other technologies to simulate the spatial thermal environment is summarized.

### 2.1. Definition of CFD-Based USTE Simulation

CFD lies at the intersection of modern fluid mechanics, numerical mathematics and computer science. Computers are used as tools, and various discretized mathematical methods are applied to perform numerical experiments, computer simulations and analyses to address various problems in fluid mechanics. CFD encompasses a variety of practical turbulence models, which can be applied to complex flow simulations in different fields. Three-dimensional mathematical models of certain areas can be established to simulate the USTE, and the airflow in this region can be numerically calculated. After establishing the input variables (e.g., climate boundary conditions and urban surface boundary conditions) and the system structure and characteristics (including control equations, discrete equations, and convergence conditions), the target variables (e.g., microclimate parameters, such as wind, temperature, humidity, and atmospheric pollution parameters) are calculated and output. Therefore, CFD has become an important approach for many researchers to obtain three-dimensional temperature fields [16]. However, the accuracy of CFD models is often limited, and verification of the simulation results can be difficult because the accuracy of the results mainly depends on the CFD parameter settings and the choice of turbulence model.

### 2.2. Introduction of CFD-Based USTE Simulation Software

The software packages currently used to simulate the USTE mainly include CFD series, ENVI-Met, AUSSSM TOOL, 3D-CAD [17], OpenFOAM, and DUTE. These packages are all suitable for simulation-based research on complex problems involving fluid flow and heat transfer [17,18]. However, the methods and models used in these packages are different. Generally, numerical simulation methods can be divided into distributed parameter methods and lumped parameter methods. Distributed parameter methods are developed based on CFD models. Through CFD theory, convection, heat conduction and radiation heat transfer in a residential area are coupled to perform thermal environment calculations in a residential area. Lumped parameter methods are simplified analysis methods that ignore the internal thermal resistance of objects. Most related studies have focused on analyses of the heat balance around buildings and the heat transfer process by simplifying the consideration of the influence of flow on heat transfer. The lumped parameter approach considers the internal temperature of an object to be independent of its spatial position and only a function of time. Common lumped parameter calculation models include the one-dimensional energy balance model, interconnected energy balance model, two- and three-dimensional dynamics models, cluster thermal time constant (CTTC) model [19,20,21], surface thermal time constant (STTC) model and the corresponding improved models. The main focus of lumped parameter methods is the airflow around a building. A building is regarded as an object with no internal temperature difference, which simplifies the influence of air flow on heat transfer. Thus, the corresponding heat balance and the average temperature over time can be calculated and analyzed.

Other CFD software packages include ANSYS Fluent, PHOENICS, ENVI-Met, CFX, Star-CD, and Star-CCM. CFD technology has the unique advantages of low cost and relatively fast speed, and all types of data can be considered; additionally, various working conditions can be simulated, and CFD models have high potential for use in sustainable design. With CFD technology, convection, heat conduction and radiation heat transfer in the spatial thermal environment in a residential area can be coupled, and the thermal environment in a residential area can be predicted. ANSYS Fluent adopts a finite volume numerical simulation method, which can achieve high convergence accuracy in the simulation of structured grids and unstructured grids [22].

ENVI-met is a three-dimensional microclimate model based on CFD and thermodynamics and was developed by Michael Bruse, University of Mainz, Germany. This software can simulate processes such as air flow around a building, heat and water vapor exchange on the ground and at a building surface, turbulence, heat and moisture exchange between plants and the surrounding environment, bioclimatology, and particle diffusion. During the simulation process, ENVI-met fully considers the instability of the outdoor microclimate [18]. However, the software adopts a simplified radiation calculation method, which requires an unsteady solution of the air flow field and many calculations. Consequently, it is difficult to optimize the analysis of blocks with complex building structures, so the turbulence model used is most suitable for the analysis of medium- to large-scale USTEs.

Ecotect is an ecological building design software that was developed by Square One in the UK [23]; it is mainly used in the program design stage and provides six powerful analysis functions: thermal environment, light environment, acoustic environment, sunshine, economic and environmental impact, and visibility functions. In addition, Ecotect includes a visual weather data analysis module, which includes the main factors that influence the architectural design. In the process of building plan design, Ecotect can be used to accurately and quantitatively analyze the results of shading, ventilation and energy consumption, and provide a quantitative basis for building design and energy savings. However, this software is mostly used for indoor thermal environment analysis, and it can calculate the temporal changes in the average indoor temperature but cannot simulate the three-dimensional spatial distribution of the temperature.

AUSSSM TOOL software originated from a revised template model (revised-AUSSSM) for the synchronous simulation of buildings, cities, and underlying surfaces [24]. At present, the software includes a user graphical interface function. The purpose of AUSSSM TOOL software is to determine various parameters in the urban boundary layer structure, such as the air temperature, heat consumption of an air conditioning system, and energy heat balance. These parameters can be used to evaluate the urban heat island effect, but at present, this program can simulate only the surface temperature of a block and not the air temperature.

Thermal design tools based on 3D-CAD can simulate the thermal balance of urban surfaces [17]. The advantage lies in considering the actual design of a space, which may include buildings, the ground surface and greenery. Users can obtain the simulated temperature of each modeled building and the ground area of specific buildings, districts, or even entire cities through calculations. To date, this software has not been effectively used to simulate and predict local air temperature and humidity.

DUTE (Design Urban Thermal Environment) software is an auxiliary design analysis tool for the urban residential area thermal environment and was developed by South China University of Technology based on the CTTC model [21]. The CTTC model is based on the heat budget of a building complex, and it is simple, effective, and suitable for the prediction and evaluation of the building thermal environment in the engineering field. DUTE is mainly used in urban planning and design, planning management, architectural design, real estate development and other fields; it can be used to quickly analyze the thermal environment in residential areas and assist in urban planning and architectural design. Moreover, this model provides a quantitative indicator for improving the thermal environment in urban residential areas, ensuring the comfort and safety of the thermal environment in residential areas, improving people’s quality of life and reducing building energy consumption.

**Figure 2:**
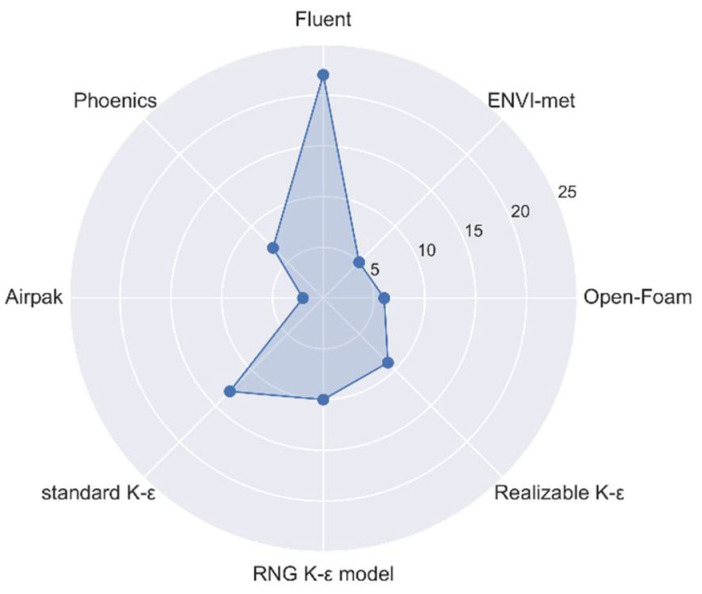
The distribution of the proportion of usage of different CFD simulation software packages and *k*-*ε* models.

At present, two CFD numerical simulation methods, ANYSYS Fluent and ENVI-Met, are widely used by researchers as the main research methods of urban space thermal environment. Bouzuidja et al. (2021) described the difference between the methods of numerical simulation in the two types of software [25]. We have summarized the main characteristics and different application fields of these methods, as shown in Table 1. According to the literature on the CFD methods used to simulate the spatial thermal environment, we performed a quantitative statistical analysis of different CFD software packages and *k*-*ε* turbulence models (as shown in Figure 2). We found that the software most used by researchers is ANSYS Fluent, followed by Phoenics. Among the different turbulence models, the standard *k*-*ε* model is most commonly used, accounting for approximately 45.7% of all use instances, and the realizable *k*-*ε* model and the RNG *k*-*ε* model are equally used.

### 2.3. CFD Numerical Simulation Models

Numerical simulation methods based on fluid dynamics are mainly divided into three types: direct numerical simulation (DNS), Reynolds-averaged Navier-Stokes (RANS) simulation and large eddy simulation (LES). DNS requires many calculations, and it is relatively difficult to apply in large-scale simulation research [17,21,22,23]. In high-Reynolds-number fluid flow problems and wall turbulence fluid flow problems, the LES method is limited because it requires hardware with a large computing capacity. Therefore, the RANS method is one of the most commonly used methods in USTE simulation research [18].

The simulation theory based on the RANS method is also called turbulence mode theory. The NS equation in the RANS model is the governing equation for the average variable of the flow field. The RANS method provides only average information on turbulence, and it can transform a turbulence problem involving unsteady flow into a steady problem for analysis. In this transformation, there will be more unknown parameters than the number of governing equations. Therefore, supplementary equations need to be added to obtain a solution.

According to the number of variables used in the calculation or the number of supplementary equations, the common turbulence models can be divided into zero-equation models (algebraic models), one-equation models and two-equation models. Among them, the *k*-*ε* two-equation model is the most used. With the development of the RANS model, the *k*-*ε* two-equation model is divided into the standard *k*-*ε* model [28], RNG *k*-*ε* model [29] and realizable *k*-*ε* model [30]. The differences among, and usage of, these three *k*-*ε* models are listed in Table 2.

- **Standard *k*-*ε* model:**

(1) $$c_{\mu }=0.09;\;\delta_{k}=1.0;\;\delta_{\epsilon }=1.3;\;c_{1\varepsilon }=1.44;{\;c}_{2\varepsilon }=1.92$$

Turbulent kinetic energy *k*:

(2) $$\frac{\partial }{\partial t}\left(\rho k\right)+\frac{\partial }{\partial x_{i}}\left(\rho ku_{i}\right)=\frac{\partial }{\partial x_{j}}\left[\left(\mu +\frac{\mu_{t}}{\delta_{k}}\right)\frac{\partial k}{\partial x_{j}}\right]+G_{k}+G_{b}-\rho \varepsilon +S_{k}$$

Turbulence dissipation rate *ε*:

(3) $$\frac{\partial }{\partial t}\left(\rho \varepsilon \right)+\frac{\partial }{\partial x_{i}}\left(\rho \varepsilon u_{i}\right)=\frac{\partial }{\partial x_{j}}\left[\left(\mu +\frac{\mu_{t}}{\delta_{k}}\right)\frac{\partial \varepsilon }{\partial x_{j}}\right]+G_{1\varepsilon }\frac{\varepsilon }{k}\left(G_{k}+C_{3\varepsilon }G_{b}\right)-C_{2\varepsilon }\rho \frac{\varepsilon^{2}}{k}+S_{\varepsilon }$$

- **RNG *k*-*ε* model:**

(4) $$c_{\mu }=0.0845;c_{1\varepsilon }=1.42;{\;c}_{2\varepsilon }=1.68;\;\alpha_{k}=1.39;\alpha_{\varepsilon }=1.39$$

Turbulent kinetic energy *k*:

(5) $$\frac{\partial }{\partial t}\left(\rho k\right)+\frac{\partial }{\partial x_{i}}\left(\rho ku_{i}\right)=\frac{\partial }{\partial x_{j}}\left(\alpha_{k}\mu_{eff}\frac{\partial k}{\partial x_{j}}\right)+G_{k}+G_{b}-\rho \varepsilon +S_{k}$$

Turbulence dissipation rate *ε*:

(6) $$\frac{\partial }{\partial t}\left(\rho \varepsilon \right)+\frac{\partial }{\partial x_{i}}\left(\rho \varepsilon u_{i}\right)=\frac{\partial }{\partial x_{j}}\left(\alpha_{\varepsilon }\mu_{eff}\frac{\partial \varepsilon }{\partial x_{j}}\right)+G_{1\varepsilon }\frac{\varepsilon }{k}\left(G_{k}+C_{3\varepsilon }G_{b}\right)-C_{2\varepsilon }\rho \frac{\varepsilon^{2}}{k}-R_{\varepsilon }+S_{\varepsilon }$$

- **Realizable *k*-*ε* model:**

(7) $$C_{1\varepsilon }=1.44;C_{2}=1.9;\;\delta_{k}=1.0;\delta_{\varepsilon }=1.2$$

Turbulent kinetic energy *k*:

(8) $$\frac{\partial }{\partial t}\left(\rho k\right)+\frac{\partial }{\partial x_{i}}\left(\rho ku_{i}\right)=\frac{\partial }{\partial x_{j}}\left[\left(\mu +\frac{\mu_{t}}{\delta_{k}}\right)\frac{\partial k}{\partial x_{j}}\right]+G_{k}+G_{b}-\rho \varepsilon +S_{k}$$

Turbulence dissipation rate *ε*:

(9) $$\frac{\partial }{\partial t}\left(\rho \varepsilon \right)+\frac{\partial }{\partial x_{i}}\left(\rho \varepsilon u_{i}\right)=\frac{\partial }{\partial x_{j}}\left[\left(\mu +\frac{\mu_{t}}{\delta_{k}}\right)\frac{\partial \varepsilon }{\partial x_{j}}\right]+\rho C_{1}S\varepsilon -\rho C_{2}\frac{\varepsilon^{2}}{k+\sqrt{\vartheta \varepsilon }}+G_{1\varepsilon }\frac{\varepsilon }{k}C_{3\varepsilon }G_{b}+S_{\varepsilon }$$

RANS can quickly simulate the flow performance characteristics of a fluid, such as the average velocity field, average scalar field and average force of turbulence. However, when the flow field contains a large-scale, unsteady vortex structure, the model struggles to converge and may not accurately simulate the flow field structure. Thus, more powerful calculation capabilities are required [31]. Kim et al. (2000) used three different *k*-*ε* turbulence models based on the RANS equation to simulate the wind field at Mount Askervein [32]. Compared with the standard *k*-*ε* and realizable *k*-*ε* models, the RNG *k*-*ε* turbulence model can more accurately calculate the average velocity and turbulence. The study by Li et al. (2006) showed that the realizable *k*-*ε* scheme is more effective than other schemes in simulating the atmospheric processes around complex terrain [33]. Antoniou et al. (2017) used LES *k*-*ε* and RANS *k*-*ε* models to simulate outdoor wind fields in complex, dense urban areas [34]. The results showed that the LES model could accurately predict the average wind speed and turbulence intensity in complex urban areas. Compared with the LES method, the RANS model yielded a 52% deviation.

In recent years, many scholars have begun to couple RANS and LES models to study complex turbulence problems [30,35,36,37]. In different study areas, RANS and LES methods are used for simulation to balance the calculation cost and simulation accuracy. However, this coupling approach has many problems when dealing with a wide range of complex flow scenarios [38]. According to many previous studies, the RANS model is characterized by poor universality, therefore, appropriate turbulence models should be selected for different research problems.

At present, many scientists have begun to couple CFD models and other models to perform simulation research on the thermal environment of urban buildings. Refs. [18,39] coupled CFD methods and building energy models (BEMs) to study the urban heat island effect at the block scale. The initial temperature of buildings was obtained through BEM simulations. Then, these results were used as the boundary conditions in CFD simulations. Based on the CFD model applied, the heat flow distribution was simulated for different geometric characteristics and architectural forms.

### 2.4. Using CFD to Simulate the USTE Based on Ground Observations

Early research on the heat island effect was mainly obtained through ground observations at meteorological stations. Lake Howard (1818) assessed the difference in temperature between urban and suburban areas in London through field observations, and first identified the urban heat island effect. The parameters measured on site mainly included surface temperature, air temperature, wind speed, humidity, and other parameters. Since then, with the development of ground observation methods, various sensors have been installed on mobile collection vehicles and airplanes to obtain thermal environment data, such as temperature, humidity, and wind speed, at different locations [40]. Johansson (2006) used a fixed weather station in the old and new cities of Fez, Morocco to conduct long-term monitoring of sites with different geometric characteristics. Short-term measurements were performed at 10 other locations to capture the spatial characteristics of the urban climate [41]. Klemm et al. (2015) used mobile stations installed on bicycles to collect data by cycling on nine streets in Utrecht, the Netherlands. The collected data was used to evaluate the physical and psychological effects of street greening on the outdoor thermal comfort of pedestrians [42]. Although the accuracy of meteorological observation data and on-site measured data is relatively high, meteorological stations and measurement points are limited in number, unevenly distributed, and spatially discrete. Thus, the regional representativeness of recorded data is relatively poor. In addition, the field measurement method is easily influenced by weather and human factors, which affect the accuracy of the data [41].

**Figure 3:**
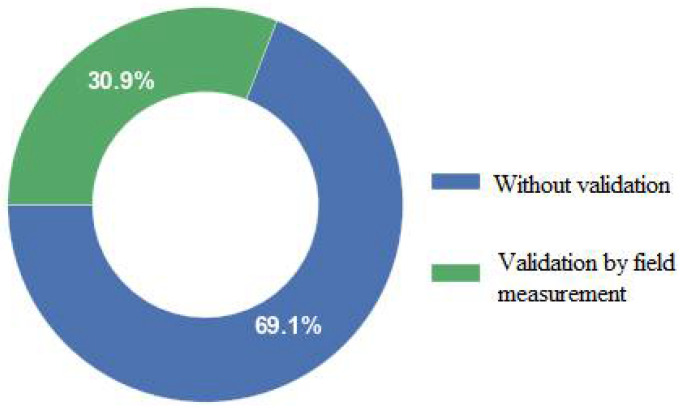
Statistical distribution diagram of the studies that combined CFD simulation and field measurements.

Few studies have simulated and studied the thermal environment based solely on on-site measured data. Typically, measured data are used to provide boundary conditions for numerical simulation studies or to validate the results of simulations and estimate the simulation accuracy [42,43,44]. Antoniou et al. (2019) simulated air temperature, surface temperature, and wind speed based on CFD methods and validated the simulation results with field-measured data. The simulation results exhibited good performance and high accuracy. The temperature deviation was ±1.35 °C, the wind speed deviation was ±0.57 m/s, and the surface temperature was ±2.31 °C [44]. Toparlar et al. (2015) used a CFD model to simulate the land surface thermal environment in a district of Rotterdam. A comparative analysis of the surface temperature retrieved from satellite image data and the results of the CFD simulation revealed that the deviation between the simulated and retrieved urban surface temperatures was 7.9% [45]. Fatima et al. (2017) simulated the temperature and wind field with a CFD approach and performed a comparative analysis based on a field measurement dataset. The average temperature deviation was 11%, and the average wind speed deviation was 19% [46]. These results indicated that the distribution of and changes in the urban space thermal and wind environment were predicted with high accuracy based on the CFD model. We calculated the number of previous CFD-based thermal environment studies that integrated field measurements and meteorological station observation data over the past two decades, as shown in Figure 3. Figure 3 shows that in early studies, the accuracy of the CFD simulation results was not evaluated, and the models were not validated. These studies account for approximately 69.1% of the studies identified. Approximately 30.9% of the studies conducted simulation result validation and accuracy evaluation; this difference may be mainly attributed to the increased availability of site data and field-measured data in recent years and the development of temperature and wind sensor technology [44].

### 2.5. USTE Simulations Integrating CFD and Remote Sensing

With the continuous development of space technology, based on advanced technologies such as geographic information systems (GIS), global positioning systems (GPS), remote sensing (RS), three-dimensional laser scanning, and aerial photography, it is possible to accurately obtain surface ecological parameters, surface temperature field information, and building geometric feature information over large areas [47,48,49,50,51]. If these data are combined with CFD technology, the USTE can be accurately estimated (as shown in Table 3). Table 3 shows some related studies that have used both CFD models and space technology, including GIS, remote sensing, and lidar technology, to simulate the USTE or wind field distribution.

The coupling of RS, GIS, lidar and other technologies with CFD can provide important data for parameter optimization in CFD models, yield precise boundary input conditions, support the accurate construction of 3D models, and substantiate the validation of CFD simulated results; additionally, this approach provides an important platform for analyses of the temporal and spatial changes in CFD simulation results [52,53,54,55,56] (as shown in Table 2). Ashie et al. (2011) used GIS technology to obtain urban building characteristic parameters and identify different types of land cover (concrete, asphalt, water, etc.), and lidar was applied to measure the height of buildings. Then, the thermal environment in 23 different urban areas in Tokyo and the spatial thermal environment distribution in the Tokyo Bay area were simulated based on CFD [54]. Hedquist et al. (2009) used temperature and humidity sensors to measure air temperature and relative air humidity, a FLIR SC640 thermal infrared camera to obtain object surface temperature information, a GPS to obtain location data, and Google Earth real-time street images to conduct 3D urban modeling. Then, the above information was input into a CFD model to simulate the distribution of, and changes in, the spatial thermal environment in Phoenix, Arizona, USA, based on different building exterior materials and different building layout conditions [40]. Some scientists have used Landsat series satellite thermal infrared image data to retrieve urban land surface temperature and perform patch clustering for the underlying surface, thus providing boundary conditions for simulating the urban heat island effect based on CFD models at the mesoscale to macroscale [56,57]. Hsieh (2016) used the frontal area index (FAI) and least cost path (LCP) indexes combined with ArcGIS to perform calculations for ventilation corridors in Tainan City, Taiwan Province, and they validated the results with CFD simulations of the ventilation corridors and thermal environment distribution [53]. Maragkogiannis et al. (2014) used lidar scanning technology and an Optech-ILRIS 3D scanner to build a 3D model of 1866 Square in Chania, Greece, and aerial remote sensing images were used to identify different land types; then, the effects of streets on the thermal environment were simulated with a CFD model [58]. In Nicosia, the capital of Cyprus, Antoniou (2019) used an FLIR-P640 thermal camera installed on a helicopter to obtain the surface temperature, an ultrasonic anemometer to measure wind speed, and an AT2 sensor to measure the air temperature. Then, the above datasets were applied in a CFD model to provide initial conditions for the simulation of the USTE [44].

It is worth noting that the above research only simply integrated remote sensing, GIS and lidar technologies with CFD models and provided accurate initialization conditions for CFD simulations of the USTE. With the development of high-resolution remote sensing technology, how to deeply couple the abovementioned technologies with CFD models, improve the use of multisource spatial data to quantitatively retrieve various urban ecological parameters, and explore building geometric features and spatial position relationship data are key problems that need to be resolved. These issues are important for the optimization of CFD models, the simulation and verification of the USTE, accuracy assessments, and analyses of USTE mechanisms.

## 3. CFD Simulations of USTE at Different Spatial Scales

**Figure 4:**
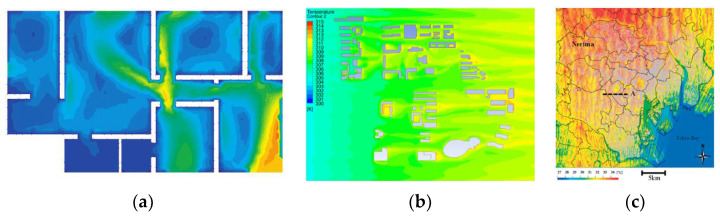
Definition and classification of scale for CFD-based simulations of the USTE: (**a**) Microscale [60]; (**b**) Block scale; (**c**) Macroscale [63].

**Figure 5:**
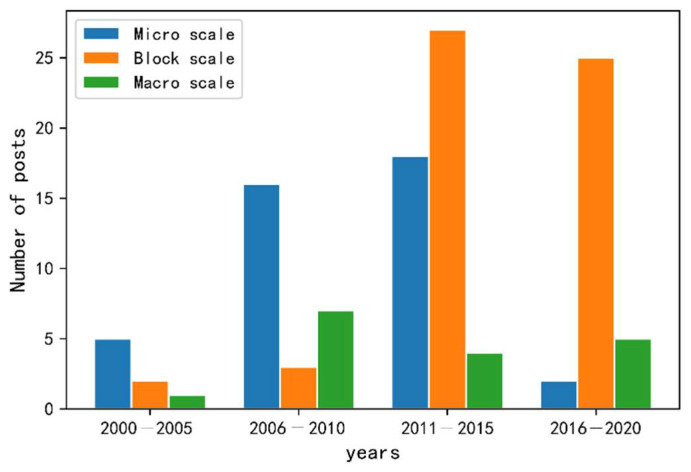
Publications focusing on three scales in CFD-based USTE simulation.

Based on previous research, we divide CFD simulations of USTEs into three scales: the microscale, block scale and macroscale [13]. CFD simulation research of single buildings and indoor thermal environments corresponds to the microscale, and the associated study areas are within a building footprint or inside the building. Study areas ranging from 100 m × 100 m to 2 km × 2 km correspond to the block scale. Study areas larger than 2 km × 2 km are defined as macroscale (see Figure 4). We performed a quantitative statistical analysis and classification of the literature on CFD simulations of the USTE at different scales in recent years (as shown in Figure 5). Many studies of the indoor thermal environment and ventilation performance of single buildings have been performed based on CFD; these studies were generally early among those identified and mainly focused on the heating, ventilation and air conditioning (HVAC) field [59,60]. Simulations of thermal environments and urban canyon wind environments at the community scale and block scale have increased rapidly in popularity over the past ten years [61]. The number of thermal environment studies based on CFD at the macroscale has increased compared to that ten years ago [62,63]. However, at the macroscale, some problems caused by the complexity of the models used, boundary condition settings and model scale have not been resolved. Therefore, the use of CFD for medium- and large-scale thermal environment research is still in the preliminary stage of exploration.

### 3.1. USTE Simulation at the Microscale

CFD has been used to simulate the thermal environment at the microscale, with a typical focus on the wind-heat environment and comfort level of a single building or building interior. The simulation results of the indoor thermal environment can be used to guide the indoor energy supply and air-conditioning design parameters [64,65,66,67,68]. Most studies in this area are focused on heating and cooling in the HVAC field and accordingly adjusted the design parameters of air conditioners and the energy required for indoor buildings [69]. Several years ago, scientists began to perform simulation studies of the outdoor wind and heat environments of single buildings based on CFD. Albatayneh et al. (2018) simulated the distribution of, and changes in, the external surface temperature of a single building based on CFD in Perth, Australia, during the Spring Festival and Winter Festival [70]. Hooff et al. (2017) focused on a single building as the research object and simulated and compared the turbulent kinetic energy results obtained based on RANS, LES and experimental methods; they found that the LES approach exhibited better performance than the other two methods, but the calculation time was longer. The results also indicated that the choice of model (RANS vs. LES) should be based on which parameter is the target parameter, noting that the use of LES will result in an increase in the computational demand [71].

### 3.2. USTE Simulation at the Block Scale

Research on CFD simulations of urban thermal environments based at the block scale with horizontal distances in the range of 200 m to 2 km has achieved valuable results [72,73]. Antoniou (2019) studied two typical urban thermal environment simulations at small and moderate regional scales and urban canyon thermal environment simulations [44,73]. Most previous CFD simulations at the block scale focused on streets, residential communities, and urban central business districts. All these areas contain almost all the basic elements of thermal environment research, including urban underlying surfaces with different physical characteristics, buildings, sparse vegetation, and water bodies [43]. CFD-based USTE research at the block scale has mainly focused on the following topics: (1) the influence and mitigation effects of vegetation, water bodies and other factors, morphological changes in these factors, and different spatial layouts on the thermal environment of local-scale urban buildings [74,75,76,77,78]; (2) the quantitative relationship between the form and layout of buildings or building groups and the local urban thermal environment [79]; (3) the layout and design of urban ventilation corridors based on the simulation results of the urban thermal environment at the block scale, as well as the planning of urban ventilation corridors to mitigate the impact on the thermal environment [61,80]; and (4) the quantitative relationship between the thermal environment and human thermal comfort indices [81].

### 3.3. USTE Simulation at the Macroscale

As the research scale has increased, the complexity of 3D modeling and CFD model simulations has also increased. Simulating the USTE at a macroscale without considering the scale of the model requires a huge amount of computer memory and high computing power. Some details can be ignored when performing 3D modeling at a macroscale compared to those at the microscale. Antoniou et al. (2019) simulated an area of 0.247 km^2^ in the city of Nicosia (the capital of Cyprus) in the middle of the Mediterranean; the calculation area was 1700 m × 1700 m [44]. Ashie et al. (2011) simulated the temperature and wind speed distributions in the coastal city of Tokyo under the influence of sea breezes; the study area was 3300 m × 3300 m [54]. Li et al. (2008) used remote sensing technology to optimize and modify the relevant parameters in CFD simulations and simulated the distribution of the urban thermal environment in Wuhan city in summer and its relationship with the main components of the underlying surface of the city, such as vegetation, water, and buildings [57]. Du et al. (2019) simulated the quantitative effects of the different layouts and geometric features of green spaces on the thermal environment of individual buildings, urban canyons, and streets based on CFD [56]. In addition, they simulated the USTE distribution in Shanghai considering the effects of aquatic vegetation and water bodies, and corresponding mitigation measures were proposed to alleviate the thermal environment in Shanghai. Hsieh et al. (2016) simulated the distribution of the wind field in Shanghai based on GIS and CFD, predicted the optimal direction of ventilation in Shanghai, and gave relevant suggestions for the planning of ventilation corridors [53].

For the above three scales, the study topics, software packages and models used in CFD research to simulate the urban thermal environment are summarized in Table 4.

## 4. Quantitative Simulations of the USTE Based on CFD Considering the Underlying Surface Dynamics

The high-temperature thermal environment in urban areas is a negative effect produced by the process of urbanization. The corresponding formation mechanism is complex and significantly regional. The main factors that influence the USTE include changes in the natural attributes of the underlying surface of the city, anthropogenic heat emissions, and air pollution. In particular, the changes in the urban underlying surface are important contributors to the urban heat island effect [6,82]. The changes in the underlying surface of a city mainly include changes in land use/land cover (LULC), the structure of the underlying surface, and the spatial geometric topological relationships among different underlying surface components [83,84]. The rapid development of urbanization has resulted in a large number of natural surfaces being replaced by urban impervious surfaces, which are the main contributors to the urban thermal environment; moreover, reductions in vegetation and water bodies, which play important roles in mitigating negatives changes in the urban thermal environment, have occurred [85].

### 4.1. Relationship between Changes in LULC and the USTE

The rapid development of urbanization has led to dramatic changes in LULC. The conversion of cultivated land, bare soil, and other natural land types into urban residential areas, industrial areas and commercial business districts has resulted in the replacement of the natural surfaces of cities with impervious surfaces. As a result, the physical properties of the underlying surface, such as the albedo, emissivity, thermal inertia, specific emissivity and thermal conductivity, have significantly changed [86,87]. These changes have led to a decline in the self-moderating ability of the urban thermal environment.

Changes in urban LULC have significantly affected the heat exchange between the surface and the atmosphere [88,89]. Research on the relationship between surface radiation and the energy balance based on CFD simulation has also attracted widespread attention from scholars [90]. Takahashi et al. (2004) used CFD numerical simulation methods to simulate the changes in the energy flux among soil, vegetation, the atmosphere and buildings and the heat exchange among various influential factors. The simulation results showed that when large numbers of green areas and water bodies are converted to urban land, the surface temperature will increase, resulting in direct changes to the USTE [43].

### 4.2. Relationship between the Underlying Surface Structure and the USTE

The changes in the underlying surface structure mainly include changes in urban vegetation coverage, water coverage, and building area caused by replacing natural permeable surfaces with urban impervious surfaces (often made of cement, asphalt, concrete and other building materials). This process will result in a positive effect for the USTE in the following two aspects. On the one hand, impervious surfaces have a stronger ability to absorb solar radiation than do natural surfaces. In addition, in the urban canopy space, part of the solar radiation reflected from the ground is absorbed in the process of reflection between buildings, which is the main energy source of the USTE [91]. On the other hand, the density of buildings in metropolitan areas is high, the number of high-rise buildings is large, and the surface roughness is high, resulting in poor urban ventilation, which causes heat to accumulate over a short period of time. If the heat cannot be appropriately discharged, the urban temperature will continue to rise and remain at a high level, thereby increasing the intensity of the USTE.

Reasonable underlying surface types and layout structures are important factors that need to be considered to alleviate the USTE [57]. Dimoudi et al. (2014) changed the roads and streets in a model from cement mixed with clay to cold materials, used CFD to simulate the temperature field distribution, and found that the surface temperature dropped by 6.5 °C [92]. According to the results of CFD simulations of wind and heat environments, Fatima et al. (2017) found that hot spots in high-temperature areas in cities have a certain correlation with low wind speeds [46]. Undeveloped areas, such as natural soil, grassland, woodland, and cultivated land areas that have been artificially developed, are characterized by good water permeability and can convert absorbed solar radiation energy into latent heat energy, which can effectively mitigate the rate of increase in surface temperature. Radhi et al. (2015) analyzed the distributions of, and differences in, the wind and heat environments between natural islands and human-made islands based on CFD simulations. Compared to uninhabited natural islands, artificial islands are mostly composed of various buildings and roads with impervious underlying surfaces, which are characterized by poor water permeability and air permeability and become hot faster than other surfaces. Under the same thermal environment requirements, the cooling capacity of human-made islands has increased by 14–26% [88]. Therefore, the proportion and layout of different underlying surfaces should be reasonably established in the relevant design and construction stages to optimize the urban wind and heat environments.

### 4.3. Relationship between the USTE and Urban Green Spaces and Waterbodies

A change in the underlying surface of a city can change the distribution of latent heat and sensible heat, potentially leading to the formation urban heat island effects [78]. Many scientists have explored the relationship between different land use types and heat island effects by integrating CFD and RS/GIS technologies [93]. Studies have shown that the types of land used for urban construction and transportation are significantly positively correlated with the urban heat island effect, and cultivated land, forest land, water bodies and other land use types are significantly negatively correlated with the urban heat island effect [55,94]. In the case of the same green area, for every 1% increase in the impervious surface area surrounding a green area, the cooling range of the green area will decrease by approximately 4.01 m [95]. Lin et al. (2007) used the regional atmosphere simulation system MM5 to simulate the urban heat island effect in Beijing; their results showed that water bodies and green spaces have a good control effect on the temperature near the ground [96]. Huang et al. (2020) simulated the wind and heat environments in Taiwan through a CFD model. They obtained quantitative indicators to improve the thermal environment and found that the green area ratio should be at least 60% to significantly improve the thermal effect [97]. Therefore, the type of urban green space, vegetation coverage, and spatial layout can all affect the urban thermal environment [42,98].

The area percentages of green space and different vegetation types affect the distribution of the thermal environment. Therefore, the quantitative impact of urban green plants on the USTE has become a hotspot in CFD-based thermal environment research in recent years. Zhang et al. (2017) found that the spatial cooling effect of a green belt becomes weaker as the area decreases [99]. When the proportion of green space reaches 20–35%, the cooling effect will be significantly enhanced, and as the green space proportion increases, the thermal comfort index will increase [100]. However, the cooling distance range of a green space generally stays between 150 m and 250 m [95]. Liu et al. (2012) simulated the impact of different vegetation types on the urban wind and heat environments based on CFD and found that shrubs hinder the flow field more-so than do trees. Because shrubs have a stronger impact on wind speed than trees, it is not advisable to position too many shrubs upwind of areas that require ventilation [101].

Under the same area conditions, various green space morphological characteristics have different effects on the thermal environment. In terms of mitigating the effects of the thermal environment, wedge-shaped and radially distributed green spaces have the best cooling effect [101]. The cooling effect of strip-shaped areas is relatively poor, but if the distribution can be well matched with ventilation corridors, these areas can also produce an excellent cooling effect [56]. A dotted green space pattern can break up high-temperature urban heat island areas and has displayed advantages in improving the thermal environment and microclimate in local areas of a city [100]. In the case of the same green area, the cooling range of the green area decreases as the shape index increases and as the perimeter of the green area increases [95].

## 5. Anthropogenic Heat Emissions and the USTE

During the rapid process of urbanization, the heat generated by human production and activities, including the heat generated by transportation, energy consumption and industrial production and the heat released by the electrical appliances used in daily life [102], has enhanced the urban thermal environment and significantly impacted the local urban climate [103]. Most urban heat island centers are located in populated residential areas, industrial areas, and urban central business districts, and the intensity of heat islands is greatly affected by human activities, making it difficult to estimate anthropogenic heat.

In the field of urban microclimate research, there are three main methods used to calculate anthropogenic heat [104], including the source inventory method, the energy balance equation method, and the numerical model simulation method. Fan et al. (2005) used the PBL model in the MM5 climate simulation package to study the thermal environment in Philadelphia and found that anthropogenic heat has a significant impact on the urban heat island effect, especially at night and in winter [105]. To introduce anthropogenic heat emissions into the CFD numerical simulation process, He et al. (2007) added anthropogenic heat to the surface energy balance equation and atmospheric heat conservation equation in a certain proportion based on a multiscale model; then, the urban thermal environment in Nanjing, Jiangsu Province, eastern China, was simulated based on the multiscale model system. They found that the contribution of anthropogenic heat source emissions to the urban heat island reached 29.6% [106]. Qian et al. (2020) generated a gridded anthropogenic heat flux benchmark dataset with a spatial resolution of 1 km based on machine learning; they found that the anthropogenic heat emissions in the city center were 60–190 W/m^2^, and the largest value of anthropogenic heat emissions in the industrial zone was 415 W/m^2^ [107]. These research results show that anthropogenic heat has become a major component of urban thermal environment research.

At present, most studies on USTEs based on CFD simulations at the block scale or macroscale do not consider the influence of anthropogenic heat emissions, which is a possible reason for the low simulation accuracy of these CFD models. However, at the microscale, a few scholars have performed research on indoor human thermal comfort considering the heat generated by the human body.

Recently, few studies have considered the influence of anthropogenic heat emissions when using CFD methods to simulate the USTE. Determining how to quantify anthropogenic heat emissions and couple them with thermal environment simulation models based on CFD is important for accurately simulating the USTE; this topic should be addressed in future research in this field. Therefore, at the block scale and macroscale, anthropogenic heat includes not only the heat emitted by humans but also the heat generated by human activities, such as transportation and industrial production. However, the estimation of anthropogenic heat is complicated. Coupling anthropogenic heat emission predictions with CFD simulation models is one of the challenges faced in the current research on CFD-based USTE simulations.

## 6. Research on Mitigation Measures for USTEs Based on CFD Simulations

The urban thermal environment is influenced by the wind environment, solar radiation, anthropogenic heat, geographic and topographic features, underlying surface thermal properties, heat conduction and thermal radiation among components of the underlying surface, and atmospheric heat convection at different altitudes [108]. To quantify and mitigate negative effects on the USTE, many scientists have performed studies on cooling cities and increasing ventilation in urban areas. Lai et al. (2019) summarized and described the cooling effects on the urban thermal environment and mechanisms of the four main mitigation strategies of changing the urban geometry, increasing vegetation, permeable surface and water bodies [109]. The influencers and mechanisms of the USTE have been analyzed from multiple dimensions and perspectives, and corresponding methods and measures have been proposed to control the USTE according to the characteristics of different cities. These measures mainly include the planning and layout of green plants and water bodies, the rational planning of building complexes, the reasonable layout and optimization of urban ventilation corridors, and the use of green and energy-saving building materials (as shown in Table 5).

### 6.1. Water Bodies for Controlling Heat in the USTE

Water bodies have a large specific heat capacity, and the rate of temperature increase is slower for water than for buildings and other surface components in cities. Under the actions of evaporation and transpiration, a water body exchanges heat with the atmosphere close to the water surface by absorbing heat and converting solar radiant energy into latent heat. Under the influence of the wind field, the cold air in the upper layer of the water body exchanges heat with the surrounding airflow, making the temperature around the water body much lower than that in areas with buildings [110].

As a heat sink, the cooling effect of a water body is related to its area, shape, and distance from a target. Yang et al. (2015) used a CFD model to simulate a river and the wind-heat environment under different building layouts. They found that when the angle between a building and a river is in the range of 45–90°, the outdoor thermal environment effect is minimal. In terms of the building height layout, a layout from low to high is better than a layout with a constant height [111]. Du et al. (2019) used a CFD model to simulate the influence of water bodies with different morphological characteristics on the heat island effect [56]. The results showed that a water body with a complicated outer contour provides a better cooling effect than a water body with a uniform outer contour. Notably, the outer surface of a complex contour is larger, which enables the water body and the air to fully exchange heat. The cooling effect that a water body can provide is also affected by the distance to the target. The closer the target is to the water body, the better the cooling effect. When a building is far from a water body, the cooling effect is weakened. Ashie et al. (2009) found that the area within a range of 100–200 m on both sides of a river is obviously affected by the cold wind that blows across the river; in contrast, the temperature in inland areas was higher [54].

The efficiency with which water bodies mitigate the effects of the USTE is affected by the combined effects of wind speed, wind direction, external shape, spatial location and area. Tominaga et al. (2015) simulated the cooling effect of water on the thermal environment of buildings based on CFD simulations. The results showed that the maximum cooling effect at pedestrian height can reach 2 °C, and the average cooling effect is approximately 0.5–1 °C. With a wind speed of approximately 3 m/s at a height of 10 m, the cooling effect caused by evaporation spreads more than 100 m downwind [112]. Hsieh et al. (2016) conducted a CFD simulation study of the ventilation environment in Shanghai and found that in urban planning, placing a water body upwind in the dominant wind direction can effectively alleviate the local heating effect [53]. Song et al. (2011) proposed a new calculation method for heat transfer and moisture transfer between water bodies and the atmosphere to study the impact of urban water bodies on the humidity and thermal environments of surrounding buildings [113].

From the current literature, the quantitative relationship between the shape of a water body and the cooling range under different wind speeds has not been clarified. In addition, the high-humidity environment formed by the evaporation of water will also affect the comfort of humans. Therefore, the quantitative relationship between these factors and comfort evaluations are important research topics related to the effects of water bodies on the urban thermal environment. When considering the mitigation of the thermal environment by a water body, the relationships among the water body, the wind speed and direction, and the building layout should be considered to control the spatial thermal field.

### 6.2. Green Space and Vegetation for Controlling the USTE

The central role of green space and vegetation in mitigating the heat island effect has attracted widespread attention from scholars [114]. The recent research has focused on the mechanism and influence of green plants in the urban thermal environment [115,116,117], the simplification of the vegetation model and its coupling with CFD [114,118], and the relationship between the USTE and various vegetation parameters, such as the relationships between the intensity of urban heat islands and vegetation index values [119,120], vegetation abundance [121], and the standardized compactness index [99].

The mechanism by which green vegetation mitigates the heat island effect is mainly reflected by the following two factors [117]. On the one hand, vegetation absorbs part of the solar radiation for photosynthesis and reflects part of the solar radiation from its leaves [92]. The temperature difference caused by trees by blocking solar radiation and absorbing energy can reach 6 °C [122]. On the other hand, the transpiration of vegetation absorbs most available heat and creates a latent heat flux, which slows the increase in temperature. Under the combined effect of tree transpiration and shading, the outdoor thermal comfort index can be effectively improved [123]. In addition, the cooling effect of green land vegetation is based on different principles at different heights. At a height of 2 m, cooling is achieved by shielding solar radiation and exchanging latent heat. At a height of 10 m, cooling is achieved by heat exchange between vegetation and the atmosphere [52].

Increasing the vegetated area and optimizing the green space structure and layout are two important measures to mitigate the USTE. Enlarging the area of an urban green space is an effective way to mitigate the urban heat island effect. The change in the leaf area index has a significant impact on the thermal comfort of the human body. The increase in the greening rate will lead to a decrease in the air temperature and the physiological equivalent temperature [124,125,126]. Lin et al. (2020) noted that for every 10% expansion in street vegetation coverage, the air temperature at the pedestrian level (height) will drop by 0.15 °C [127]. However, when the built-up area or urban land area is limited, it is not economical to rely on increasing the green area to alleviate the thermal environment problem. The most important task is rationally optimizing the structure and pattern of the green area in a limited region to achieve maximum utility. Du et al. (2019) found through CFD simulation that the layout of green vegetation with complex shapes has the best cooling effect in alleviating the effects of the thermal environment [56].

When seeking to control the thermal environment based mainly on vegetation in specific areas, various factors, including the proportion of green area, the type and spatial layout of greenery, the coordination and layout of green buildings and the wind field, must be comprehensively considered to achieve the best mitigation efficiency. To reduce the USTE, most research shows that an increase in urban green space is more effective than other surfaces with high albedo, but the research of Yuan et al. (2017) found that excessive greenery will itself have a negative effect on the microclimate of the city by reducing ventilation [128]. In addition, climate factors are also an important influencing factor of USTE [129]. Alexandri and Jones (2008) developed a comprehensive simulation study of USTE, their results depicted that a greater reduction can be achieved in urban air temperature under a hotter and drier climate through using a green wall and green roof. They also found that, from the simulation, the same configuration of green wall and green roof, the reduction of air temperature in Moscow, Russia was below 4 K, while the value of Riyadh, Saudi Arabia, was above 11 K [129,130].

### 6.3. Building Layout for Controlling the USTE

The layout of the urban structure greatly influences the urban heat island effect [131,132]. Ali et al. (2016) applied CFD simulations in urban planning and landscape design at the scale of individual buildings and communities [133]. During urban planning, the spacing and orientation of buildings are calculated based on meteorological data. The overall layout and orientation of buildings generally conform to the prevailing wind direction in summer. This design can enhance the ventilation performance of buildings, thereby making the heat distribution more even because the wind can effectively dissipate heat. Thus, creating potential ventilation corridors is important for improving a USTE. Lin et al. 2017 introduced the concept of heat balance, expounding the influence of urban built environment factors on each component of energy flux (including net radiative flux, anthropogenic heat, convective (or turbulent) sensible heat flux, latent heat flux, stored energy, and net horizontal heat advection). Based on the perspective of urban geometry (Including land use intensity, building form, canyon geometry, space enclosure and descriptive characteristics) and vegetation, the impact of these influencing factors on the urban thermal environment on pedestrian height (at pedestrain level) is quantitatively studied [134]. Chen et al. (2004) simulated the outdoor wind and heat environments based on CFD and found that the outdoor temperature in residential areas is closely related to the architectural layout of these areas [135]. The downwind temperature in residential areas was higher than that in other areas, and the thermal environment was poor. Yang et al. (2011) found that the building layout, building density and green space proportion are the main factors that influence the thermal environment in residential areas during the day [136].

The number of buildings and the height of buildings are important factors that affect the urban wind and heat environments. In the use of CFD to simulate the thermal environment of buildings of different densities, it was found that increasing the number of buildings and reducing the height of buildings are conducive to creating a good wind environment [137]. Yang et al. (2015) simulated aspects of the residential outdoor environment (such as temperature, humidity and wind speed) in Shenzhen city, southern China, under different architectural layouts based on CFD technology [111]. The effects of a river and building layout on the outdoor thermal environment around riverside residential buildings were explored. Notably, when the vertical projected area of a building facing the prevailing wind was reduced, a ventilation and cooling corridor was created for winds in the direction of the river. In coastal cities, where high-rise buildings have low building ratios, sea breezes can flow through large spaces between buildings to lower the temperature [54]. Therefore, to a certain extent, it is possible to reduce the building ratio by establishing high-rise buildings to effectively reduce the ambient temperature and control the thermal environment [138].

The aspect ratio of buildings is another important factor that influences the urban thermal environment. Specifically, solar radiation plays a leading role in influencing the energy budget of buildings with different aspect ratios. As the aspect ratio increases, the effects of longwave radiation and heat flow conduction are continuously improved. Due to shading effects, the differences in the temperature distribution in space increase. The average radiant temperature can be 10 °C higher than the air temperature, and the radiant temperature in the direct sun area can reach up to 70 °C. When the aspect ratio reaches 2, sensible heat exchange is more obvious than heat conduction [139] because as the height of the building increases, the solar radiation cannot reach the average height of the street or even the ground, radiation is reduced, and heat flow conduction increases.

### 6.4. Green Building Materials for Controlling the USTE

Research on the use of green building materials to control the thermal environment has shown that the selection of high-reflectivity building materials and pavement materials for the exterior walls and roofs of buildings has a significant effect in alleviating severe thermal environmental conditions [140,141]. For example, placing green vegetation on roofs has a positive effect on temperature, wind speed, and humidity [122]. Ferrari et al. (2020) performed CFD-based simulations of the wind-heat environment and found that the combination of high reflectivity and water-permeable pavement materials can reduce the surface temperature and create a comfortable thermal environment. In the case of low wind speeds, the use of high-reflectivity white asphalt compared to black asphalt can reduce the air temperature at a height of 1.5 m by 5 °C [86].

### 6.5. Ventilation Corridors for Controlling the USTE

Based on the wind field and USTE from CFD simulations, planning an urban ventilation corridor and analyzing the corresponding mechanism are hotspots of current research. The use of CFD simulations to accurately predict the wind environment in urban street canyons has achieved good performance [61], as demonstrated by wind tunnel tests. Shou et al. (2018) combined CFD and GIS technology to quantitatively study potential ventilation corridors in Changchun city [142]. Hsieh et al. (2016) used the FAI and LCP to predict city ventilation corridors by using ArcGIS and CFD technology to simulate and confirm the direction of ventilation corridors [53]. Ng et al. (2011) conducted a study in an area with low roughness to estimate urban ventilation corridors [143].

The ventilation environment can be adjusted to mitigate thermal environment problems. Good ventilation can reduce the heat accumulation in the center of an urban heat island, alleviate the urban heat island effect [117], and improve the comfort of the thermal environment [144]. Wind is the medium for heat exchange between a cold source and a heat source, and the main mechanism in alleviating the thermal effect is reflected by the following two factors. On the one hand, wind can effectively dissipate heat; on the other hand, wind can evenly distribute the cold air generated by water evaporation and vegetation transpiration. To mitigate the heat island effect, a reasonable ventilation corridor can be specifically designed according to different types of land use. Cold sources, such as green space and water bodies, should be built in the upwind areas of ventilation corridors, and the air will then flow through the low-temperature areas to cool the areas downwind.

## 7. Current Problems and Outlooks

We have identified the current research status of urban thermal environment simulation based on CFD models. First, the current status of numerical CFD simulation methods is summarized. Second, the integrated simulation of the thermal environment with remote sensing, GIS and lidar technologies combined with CFD models is summarized. Third, from the perspective of spatial scale, the scope of the urban thermal environment is described. Fourth, the relationship between structural changes in the underlying surface and the urban space temperature field is explained. Fifth, CFD-based research on the influence of anthropogenic-derived heat in the thermal environment and the quantification of this effect are described. Finally, research on measures and methods related to the USTE is summarized.

The problems that currently exist in CFD research mainly include (1) the parameter settings for urban boundary conditions are idealized, (2) the simplification of 3D land cover modeling does not reflect the actual conditions and targets, and (3) the accuracy of CFD model simulations is often unsatisfactory. We believe that future research involving CFD-based simulations of the USTE should focus on solving the following problems.

(1) CFD should be further coupled with remote sensing, GIS, lidar and other technologies and combined with field observation data to conduct USTE simulation research in the future. To build accurate three-dimensional models based on GIS, lidar and remote sensing data that effectively match the actual surface conditions, the initial boundary conditions should be determined. The retrieval of urban surface ecological parameters based on high-resolution remote sensing data can be used to optimize the input parameters of CFD models. Remote sensing data can be used to retrieve the surface temperature and field observation data to verify CFD simulation results. These factors are important for improving the accuracy of CFD model simulations and verifying results.

(2) Obtaining an accurate and reasonable-scale model of a city or building complex is very important when performing CFD simulation research involving the USTE at moderate and large scales, and this topic should be a hotspot of future research. If the construction of large-scale land surface models based on CFD is too simplistic, the accuracy of the simulation results will be affected. The simulation of large-scale spatial thermal environments will require considerable computing power if a full-scale model is used. For example, Ashie et al. (2011) used a supercomputer (named the “Earth Simulator”) to simulate the wind and heat environments in the 23 wards of Tokyo and the Tokyo Bay area. To reduce computational consumption and computational costs [54], the issue of scale must be considered. Pan (2020) developed a scale model to obtain the urban wind environment based on a CFD simulation method using similar criteria, compared it with the results of CFD full-scale simulations, and found that the error between the two approaches was within 5% [145]. This study provides a reference for simulating and predicting the thermal environment in large-scale areas at a low computational cost.

(3) The coupling equations for anthropogenic heat emissions and CFD models to simulate the USTE is a challenge and should be further explored in future research. The quantification of anthropogenic heat emissions can improve the accuracy of CFD model simulations. The urban population continues to increase, and energy consumption and anthropogenic heat emissions from transportation have become sources of heat that must be considered in addition to solar radiant heat in summer. The increase in anthropogenic heat emissions has had a significant impact on the thermal environment. Determining how to accurately quantify anthropogenic-derived heat emissions and couple them with CFD simulations is important for improving the accuracy of thermal environment simulations.

(4) Coupling vegetation as a porous medium into CFD simulations is an important direction for future explorations of the mechanism of vegetation in mitigation heat levels in the thermal environment. Vegetation-based cooling is a very complex physical and biological heat transfer process that involves not only the transpiration and photosynthesis of plants but also the mutual conversion of solar energy and chemical energy [146]. Many scientists have analyzed cooling mechanisms at the microscopic level [147,148]. This microscopic method is difficult to combine with CFD simulations to study the urban thermal environment at the mesoscale and macroscale. At present, many scholars have found that adding vegetation as a porous medium to CFD simulations [43,114,118,149] can improve the results of microscale and mesoscale simulations. However, considering vegetation in CFD models at the macroscale (simplifying the heat transfer process and optimizing parameters based on large-scale vegetation heat transfer mechanisms) is still an important and difficult task to consider in future research.

(5) Future research should consider the effects of water bodies on the thermal environment in CFD multiphase flow simulations. The mitigation effects of water bodies on the thermal environment are very obvious. From a microscopic perspective, the transformation of different phases occurs when a water body exerts a controlling effect on the thermal environment. Therefore, the use of multiphase flow to simulate the cooling mechanism of water bodies at the microscale is a potential future research direction. However, in large-scale CFD simulations, if evaporation-based heat absorption, flow, heat transfer and other comprehensive effects are considered, high computing power will be needed. Therefore, exploring the comprehensive effects of water on the thermal environment by simplifying water heat exchange processes and physical models while meeting the relevant accuracy requirements should be a focus of future research.

## Figures and Tables

**Table 1:** The characteristics of ANSYS fluent and Envi-met for simulation of USTE and their merits and disadvantages.

Main Software	Disadvantages	Merits	Application Scale
ANSYS Fluent	1. Many more types of datasets are required, and the setting method for vegetation is more complicated. Users are needed to define the vegetation model and its functions. 2. High requirements for numerical methods and fluid mechanics knowledge. The model and grid processing need to balance the calculation ability and calculation efficiency of computer. 3. CFD simulation requires high-resolution urban geometric models, knowledge of boundary conditions and sufficient computing resources. 4. Complex 3D cases require a higher level of CPU and computer resources to run, and it needs to be used by trained professionals.	1. It provides a variety of airflow turbulence models. 2. It can achieve most of the needs in micro-scale and block-scale research. 3. It is basically suitable for fluid mechanics problems coupled with heat and radiation transmission.	Small scale or block scale
ENVI-Met	1. Mutual reflection is not considered when calculating short-wave radiation; while long-wave radiation takes into account the average temperature. Therefore, it is difficult to assess the local effects of surface temperature changes (Lauzet et al., 2019) [26]. 2. There are stability problems when simulating curved urban canyons or adjacent communities. 3. The use of software is not provided for free. According to different uses (such as education, research or industry) there are different prices. 4. Only one turbulence model Yamada and Mellor E-ε (Yamada and Mellor, 1975) [27] is provided (Toparlar et al., 2017) [13].	1. Mainly used for the simulation of urban space thermal environment at medium and macro scale (including urban scales). 2. Almost all meteorological parameters (including temperature, relative humidity, wind speed and solar radiation) can be predicted. 3. Simulate and predict complex surface vegetation-air interactions in urban environments.	Mid/macro-scale or urban scale

**Table 2:** Differences among and usage of different K-ε models.

*k*-*ε* Models	Difference and Usage
**Standard *k*-*ε***	This model is relatively widely applied, and relatively few calculations are needed. The simulation effect is poor for complex flows, such as in large-curvature, strong-pressure-gradient, and swirling problems. The standard κ-ε model assumes that the flow is completely turbulent and that the influence of molecular viscosity can be ignored; therefore, it is only suitable for the simulation of completely turbulent flow processes [28].
**RNG *k*-*ε***	This model can be used to simulate moderate -complexity flows, such as jet impingement, separation flow, secondary flow, and swirl flow. The RNG *k*-*ε* model assumes that the vortex viscosity is isotropic [29].
**Realizable *k*-*ε***	This model is almost the same as the RNG *k*-*ε* model and can also be used to simulate the circular hole jet problem. The prediction of the divergence ratio of flat and cylindrical jets is more accurate with this model than with the RNG model. Additionally, this model yields good performance in the simulation of rotating flow, boundary layer flow with a strong reverse pressure gradient, flow separation and secondary flow [30].

**Table 3:** Combinations of CFD and space technologies applied to analyze USTEs.

Space Technology	Content	Typical Reference
**GIS**	Underlying surface and its classification	Chen 2018; Hsieh 2016; Ashie 2009; Hassan Radhi 2013
	Wind corridor calculations	Hsieh 2016
**RS**	Retrieval of urban ecological parameters related to CFD modeling Underly surface classification	Liu 2012; Li 2016; Chen 2018; Li 2008; Yi 2018; Yazhou Zhang 2017
	Land surface temperature (LST) determination Block clustering based on LST	Du 2019; Antoniou 2019; Gülten 2016; Synnefa 2011; Hedquist 2009
**Lidar**	3-D geometric modeling of buildings	Li 2016; Ashie 2011; Maragkogiannis 2014

**Table 4:** Summary of typical studies at three different scales.

Scale	Focus	Typical Literature	Software	Turbulence Model
**Microscale**	HVAC field, indoor thermal environment	Yan 2009; Ma 2019; Qin 2010; Lu 2018	ANSYS Fluent	RNG *k*-*ε*
**Block scale**	Influence of vegetation on the USTE	Liu 2012; Wang 2019; Gülten 2016; Dimitris 2017; Kubilay 2019; Gromke 2015; Du 2019	ANSYS Fluent OpenFOAM	RNG *k*-*ε* Standard *k*-*ε* Realizable *k*-*ε*
	Influence of waterbodies on the USTE	Tominaga 2015; Montazeri 2017	ANSYS Fluent	RNG *k*-*ε* Realizable *k*-*ε*
	Influence of underlying surfaces on the USTE	Chen 2018	Phoenics	Standard *k*-*ε*
	Effects of the building form, building layout and building materials on the USTE	Schrijvers 2020; Synnefa 2011; Yang 2015; Peng 2017; Ferrari 2020	Phoenics	Standard *k*-*ε*
	Planning and simulation of ventilation corridors	Tominaga 2008; Antoniou 2017	ANSYS Fluent	Standard *k*-*ε* Realizable *k*-*ε*
	Thermal environment simulation and thermal comfort	Takahashi 2004; Li 2016; Antoniou 2019; Maragkogiannis 2014; Vuckovic 2018; Toparlar 2015; Fatima 2017; Santamouris 2018; Allegrini 2017	ANSYS Fluent Phoenics ECOTECT OpenFOAM	Realizable *k*-*ε* Standard *k*-*ε*
	Anthropogenic heat	Yuan 2020	ANSYS Fluent	SST k-ω
**Macroscale**	Planning and simulation of ventilation corridors at the city scale	Ashie 2011; Hsieh 2016	WindPerfect Phoenics	
	Relationship between surface parameters and the USTE at the city scale	Li 2008		
	Influence of aquatic vegetation on the USTE	Du 2019; Zhou 2016	ANSYS Fluent	Standard *k*-*ε*
	Analysis of regional thermal environment effects	Ashie 2011; Piroozmand 2020	OpenFOAM	Standard *k*-*ε*

**Table 5:** Research on control methods for the urban thermal environment based on CFD.

Mitigation Measures	Focus	Typical Reference
Waterbodies	Water area and proportion	Tominaga 2015
	Water morphological characteristics	Montazeri 2017
Vegetation and green spaces	Vegetation type	Gülten 2016; Dimitris 2017; Gromke 2015
	Vegetation form and layout	Liu 2012; Li 2016; Du 2019; Vuckovic 2018; Zhou 2016; Lin 2019
	Green area and proportion	Wang 2019; Huang 2020; Liu 2019
Building layout and materials	Building layout	Schrijvers 2020; Peng 2017; Liu 2020; Allegrini 2017
	Building materials	Maragkogiannis 2014; Dimitris 2017; Gagliano 2017; Ferrari 2020; Santamouris 2018; Allegrini 2017; Dimoudi 2014; Priyadarsini 2008
	Planning of the underlying surface	Chen 2018; Li 2008; Hsieh 2016; Kubilay 2019; Yi 2018; Zhou 2018
Planning and design of ventilation corridors	Planning and design of urban ventilation corridors	Ashie 2011; Hsieh 2016; Wu 2009; Tominaga 2008; Antoniou 2017; Allegrini 2014
